# The Risk of Joint and Neck Injuries in Mixed Martial Arts—Grappling and Submission Techniques in Professional Fights

**DOI:** 10.3390/jcm14217467

**Published:** 2025-10-22

**Authors:** Katarzyna Mańka-Malara, Maciej Trzaskowski

**Affiliations:** 1 Department of Orthodontics, Medical University of Warsaw, 02-091 Warsaw, Poland; 2 Department of Prosthodontics, Medical University of Warsaw, 02-091 Warsaw, Poland; maciej.trzaskowski@wum.edu.pl

**Keywords:** injury prevention, traumatology, joint injury, sports medicine, combat sports, martial arts

## Abstract

**Background/Objectives**: Mixed martial arts (MMA) is a popular combat sport combining techniques from various martial arts. The most common injuries in MMA are head, wrist, and hand trauma. However, there is limited research on injuries specifically related to submission techniques prevalent in this discipline. The use of submission techniques, particularly rear-naked chokes and arm locks, may potentially lead to serious injuries. **Methods**: Submission techniques from 2488 fights from Ultimate Fighting Championship (UFC) were analyzed. Fights from all numbered events between 2000 and 2021 were analyzed, focusing on techniques that lead to fight termination, such as chokes and armbars. Official fight statistics, with additional video verification, were used. The results were statistically analyzed with comparisons between weight categories and sex. **Results**: Submissions end 18.7% of all MMA fights. The most common submission technique was rear-naked choke, accounting for 68.6% of all submissions. The arm and hand concentrated submission techniques, including the armbar, accounted for 27.1% of all submissions. Female fights were, on average, one and a half minutes longer than male bouts and had significantly fewer submission attempts. **Conclusions**: MMA fights frequently end by submission, and many submissions involve techniques that put considerable stress on the joints and cervical region. Fighters and referees need clear protocols to swiftly recognize when a submission is causing excessive risk.

## 1. Introduction

Mixed martial arts (MMA) is a relatively young combat sport, but its high popularity warrants special attention. Although MMA was established around the 1990s, it has rapidly gained a high number of practitioners of all ages as well as fans worldwide, with new injuries and psychosocial consequences emerging [1,2,3]. During the fight, various techniques are allowed, which constitutes a discipline with a high risk of different types of injury [1,4,5,6,7]. According to Ross et al. [1], in 57% of MMA fights, athletes sustain at least one injury. The injury rate is higher in professional fights than in amateur fights—68% to 51%. According to a meta-analysis of MMA injuries, the region most at risk is the head (66.8–78.0%), followed by wrist and hand (6.0–12.0%) [7]. The risk of head trauma and concussion has been a subject of many reports in the literature [8,9,10,11]. However, there is a limited number of studies concerning the risks associated with the submission techniques in this combat sport. During submission, a fighter is exposed to a technique causing serious pain or discomfort and decides that they cannot withstand it any longer. Most submission techniques concentrate on joints causing injury or the neck to choke the opponent. A survey study conducted among Brazilian jiu-jitsu (BJJ) fighters states that 60% of them are injured during competitions [12]. The primary reported injuries were sprains and strains to the fingers, upper extremities, and neck. Grappling and submission techniques from BJJ are allowed in MMA fights.

Analysis of fight results and comparison between weight categories is beneficial for creating training strategies and injury prevention plans. Understanding the successful performance in a professional fight is needed for establishing an ideal physiological profile for MMA success. This sport involves intermittent activity with periods of engagement that include both high forces and high-velocity actions [13]. Fights are scheduled for three to five rounds, each lasting five minutes. Combining both striking and grappling techniques involves different physiological requirements for the athlete—high isometric and dynamic strength is needed for grappling, while for striking, a rapid expression of force is required [14]. The evaluation of submissions ending professional MMA fights may provide insight into the potential successful fight profile in this discipline—both in terms of sports performance and athletes’ safety. Additionally, the inclusion of female fighters in the evaluation is necessary—most previous studies have focused solely on males, and potential gender differences have not been adequately considered. The winning strategies in combat sports are often closely tied to injuries; thus, the aim of this research was to calculate the number of different submission techniques that ends fights in professional mixed martial arts matches and estimate the risk associated with them.

## 2. Materials and Methods

### 2.1. Study Design

Fight statistics from all professional 2488 MMA fights (169 female and 2319 male) that took place on Ultimate Fighting Championship (UFC) numbered events between 2000 and 2021 were evaluated. From those twenty-one years of competitions, only 24 fights were excluded from the database as their status was “no-contest” due to the use of illegal techniques or failed drug tests. The official fight statistics were used. The UFC fight data are coded by professional referees and published on the website http://ufcstats.com/statistics/events/completed (accessed on 27 July 2025). The fight result is coded as knockout/technical knockout (KO/TKO), submission, decision (unanimous, majority, or split) disqualification, or overturned. Additionally, it includes data on the round in which the fight ended, the number of rounds, the knockout technique, the number of strikes (punches into the opponent), takedowns (technique that resulted in throwing opponent to the ground), submission (technique resulting in the stop of the fight due to the resignation of a fighter or a referee decision), and control time. Official fight statistics do not include information about the injuries sustained during the fights. However, a fight ending due to submission means that it posed a potential risk of an injury to the opponent, as the referee stopped the fight, the fighter decided to surrender, or could not continue the fight. All official fight statistics were manually rewritten to create a database for further evaluation. The frequency of submissions with a special focus on the type of technique used was noted. The number of different choking techniques was also evaluated, as submission techniques aiming at restricting an opponent’s cerebral blood flow or limiting airflow through the trachea were considered. Additionally, a group of submission techniques concentrating on the arm and hand area was selected. In this group, grappling maneuvers are designed to control or incapacitate an opponent by applying joint locks or hyperextension forces to the elbow, shoulder, or wrist. The rationale for introducing this classification was to assess the risk of injury to specific anatomical structures that could be potentially damaged as a result of the applied technique. If a description of the final submission technique was unclear, the classification was verified based on the video registration of the fight. The verification was conducted independently by two researchers, and their assessments were subsequently compared, with no discrepancies identified.

### 2.2. Statistical Analysis

Statistical analysis was conducted in SPSS 27. The comparisons were made both between males and females as well as across different weight categories. Because the database included fewer female fights and the number of fights varied among weight categories, non-parametric methods were applied. Specifically, Mann–Whitney tests were conducted for interval and ratio variables comparing male and female fights. For comparisons between weight categories, the Kruskal–Wallis test was used. If the dependent variables were nominal, chi-square tests were used. In all analyses, *p*-values < 0.05 were considered significant. For multiple comparisons following the Kruskal–Wallis test, the Bonferroni correction for multiple comparisons was applied. Finally, to test a monotonic relationship between weight categories and fight duration, a non-parametric Spearman correlation was calculated between the weight of the fighters (the upper limit for the category) and fight duration.

## 3. Results

There were 2488 fights included in the evaluation—169 female fights and 2319 male. Fights were scheduled for three to five rounds, with a longer duration for the title fight. Notably, fight times differed significantly between female bouts (*n* = 169; M = 12 min 5 s; SD = 6 min 16 s) and male bouts (*n* = 2319; M = 10 min 27 s; SD = 6 min 11 s; Mann–Whitney’s U = 225,230, *p* < 0.001)—female fights were on average one and a half minutes longer. In addition to duration, outcomes also differed statistically between male and female fights (Table 1, Chi^2^(3) = 11.83, *p* = 0.008). Specifically, women’s fights were relatively more likely to end due to a split decision or unanimous decision, and less likely to end in a knockout or technical knockout. Finally, the 17 men’s fights with the fewest reasons for stoppage (majority decision: 6 fights; disqualification: 10 fights; technical decision: 1 fight) were omitted from the analyses.

Fight results statistically significantly different between title fights and non-title fights (Table 2, Chi^2^(3) = 15.41, *p* = 0.001). There were relatively more endings due to KO/TKO in title fights than in non-title fights. Table 3 shows the relationship between fight results and weight categories. Due to the low numbers of catchweight and women’s featherweight fights, these categories were not included in the comparison.

Fight results differed among weight categories. The greatest variation occurred in the frequency of fight stoppage by KO/TKO, with an average of 36% of fights being terminated in this manner, and percentages ranging from 18% in the flyweight to 45% in the light heavyweight division. In contrast, the percentages of fights ended by submission and by split decision were more similar. For submissions, the average was 19% with ranging from 9% in the featherweight to 23–24% in the middleweight and welterweight categories. Split decision endings ranged from 0% in the women’s featherweight to 18% in the women’s bantamweight category. Unanimous decision rates varied as well, from 23% in the heavyweight category to 50–53% in catchweight, featherweight, and women’s flyweight categories.

To further examine these relationships, an additional analysis of weight category and fight results in men’s fight was conducted using a rang correlation analysis. Results showed that the lower the weight limit in a category, the more frequent fight ending due to unanimous decision (rS(8) = −0.98 ***, *p* < 0.001) and split decision (rS(8) = −0.80 ***, *p* = 0.018), while interruptions due to KO were less frequent (rS(8) = 0.92 ***, *p* < 0.001). The frequency of fight stoppage by submission was unrelated to weight category (rS(8) = −0.08, *p* = 0.844).

Table 4 presents the proportion of title fights and non-title fights in the analyzed database, categorized by the gender of the athletes. The proportion of title fights and other fights differed significantly between male and female fighters (Chi^2^(1) = 17.90, *p* < 0.001).

Fight durations differed between weight categories (Table 5; Kruskal–Wallis = 106.96, *p* < 0.001). Specifically, fight times in the heaviest categories—heavyweight and light heavyweight were significantly shorter than those in most other categories. Furthermore, a weak but statistically significant relationship was found—the higher the weight category in the men’s divisions, the shorter the fight time (rS(4604) = −0.19 ***, *p* < 0.001). In contrast, an analogous relationship did not occur in women’s bouts (rS(338) = −0.11, *p* = 0.051).

The duration of fights terminated due to submission was also evaluated. Analyses were conducted for two indicators: the number of rounds and the duration of the fight (in seconds). The majority of fights ended by submission were one-round (Table 6; 52.20%), followed by two-round fights (30.30%), and then three-round fights (15.60%). The percentages of fights completed by submission with each number of rounds were not statistically significantly different between men’s fights and women’s fights (Chi^2^(4) = 4.53, *p* = 0.339). Men’s and women’s fights completed by submission were not statistically significantly different in duration (men’s fights: M = 6 min 9 s, SD = 4 min 17 s; women’s fights: M = 6 min 35 s, SD = 5 min 3 s, U = 6789, *p* = 0.662).

Title fights lasted more rounds than non-title fights (Table 7; Chi^2^(4) = 89.26, *p* < 0.001)—among title fights ended by submission, relatively fewer fights than non-title fights were ended in the first and third rounds. Title fights ended by submission were longer (M = 9 min 27 s, SD = 6 min 23 s) than non-title fights (M = 5 min 49 s, SD = 3 min 44 s; U = 12,552.5, *p* < 0.001). Table 8 shows the percentages of fights ended by submission in a given number of rounds by weight category of fighters. Due to the low number of fights ended by submission in some weight categories and in a portion of rounds, it was not possible to conduct a formal analysis of the differences between weight categories in terms of the number of rounds in which fights were ended by submission.

Table 9 presents the durations of fights ended by submission, categorized by according to weight category. These times were not statistically significantly different between weight categories (U = 18.652, *p* = 0.097).

The fight control time was compared between fighters who won the fight and those who lost the fight. Fighters who won the fight controlled the fight for an average of 193.8 s (3 min 14 s; SD = 3 min 34 s), while fighters who lost the fight controlled the fight for 82 s (1 min and 22 s; SD = 1 min 55 s). The difference in fight control time was statistically significant (t(4974) = 22.94, *p* < 0.001). The same difference occurred in men’s bouts (F1: M = 3 min 15 s, SD = 3 min 36 s; F2: M = 1 min 21 s, SD = 1 min 54 s; t(4636) = 22.57, *p* < 0.001), and in women’s bouts (F1: M = 3 min, SD = 3 min 13 s; F2: M = 1 min 42 s, SD = 2 min 9 s; t(336) = 4.36, *p* < 0.001). The control time of the fight, categorized by weight and whether the fighter won or lost, was analyzed using a two-factor analysis of variance (category × fighter) (Table 10). The analysis found a significant effect of weight category (F (94,487) = 4.01, *p* < 0.001), a significant effect of fighter (F (14,847) = 264.44, *p* < 0.001), and a significant effect of the interaction of fighter and weight category (F (94,847) = 3.24, *p* < 0.001). Post hoc analyses showed that fight control time by athletes was significantly longer in the welterweight category than in the bantamweight, heavyweight, and light heavyweight categories. Differences in fight control time between athletes were greatest in the flyweight and welterweight categories.

Table 11 includes all submission techniques ending fights in different weight categories, with a calculation of the total occurrence in the analyzed fights. The most common submission technique was rear-naked choke, which ended 154 fights. Others that occurred repetitively included guillotine choke—89, armbar—63, triangle choke—35, arm triangle—26, and kimura—21 fights. For further analysis, 462 fights were selected due to the submission by different types of chokeholds or techniques concentrating on the hand or arm. A total of 317 fights were ended by chokes (68.6% of fights ended by submission), and 125 fights were ended by hand/arm techniques (27.1% of fights ended by submission) (Table 11). In men’s fights, 69.9% of fights ended by submission were ended by chokes, and 25.5% of fights ended by hand/arm techniques. In women’s fights, both fights ended by chokes and fights ended by hand/arm techniques were 50% of all fights ended by submission. The differences between the groups were statistically significant—significantly more male fights were ended by chokes (Chi^2^(1) = 5.16, *p* = 0.023), and significantly more female fights were ended by hand/arm techniques (Chi^2^(1) = 18.39, *p* < 0.001). In title fights, fights ended by chokes accounted for 66.7% of fights ended by submission, while fights ended by hand/arm techniques made up 33.3% of fights ended by submission. In non-title fights, those ended by chokes were 68.8% of fights ended by submission, and fights ended by hand/arm techniques accounted for 26.4%. The differences between the title fights and the other fights were not statistically significant (chokes: Chi^2^(1) = 0.088, *p* = 0.767; hand/arm techniques: Chi^2^(1) = 0.995, *p* = 0.318). Table 12 presents the percentages of fights ended by chokes and hand/arm techniques categorized by weight. Some weight categories were excluded from the analysis due to a small number of submissions by chokeholds or hand/arm techniques.

The number of submission attempts was compared between the sexes. Women’s fights had significantly fewer submission attempts (M = 0.32, SD = 0.71, *n* = 338) than men’s fights (M = 0.45, SD = 0.91, *n* = 4638; U = 738,803.5, *p* = 0.023). Similar gender differences were not found when the sexes were compared within the same weight category. The number of submission attempts differed significantly between weight categories (Table 12; U = 52.43, *p* < 0.001). Statistically significant differences were found for pairs: light heavyweight–welterweight (adjusted *p* = 0.001); light heavyweight–middleweight (adjusted *p* = 0.003); light heavyweight–lightweight (adjusted *p* < 0.001); heavyweight–welterweight (adjusted *p* = 0.034); heavyweight–lightweight (adjusted *p* = 0.002). No linear relationship was found between the weight limit in a category and the number of submission attempts (rS(4942) = −0.03, *p* = 0.077) (Table 12).

The percentage of defended takedowns was also compared between the sexes. Women’s fights (M = 28.4%, SD = 36.7%, *n* = 338) did not differ significantly in the percentage of defended takedowns from men’s fights (M = 27.5%, SD = 36.3%, *n* = 4638; U = 793,157.5, *p* = 0.690). No differences were also found within any of the weight categories. No differences were found in terms of the percentage of defended takedowns between title fights (M = 24.8%, SD = 34.8%, *n* = 522) and other fights (M = 27.9%, SD = 36.5%, *n* = 4454, U = 1,121,163, *p* = 0.147). The percentage of defended takedowns differed significantly between weight categories (Table 13; U = 23.49, *p* = 0.024). Despite the significant category effect, no differences were found between pairs of weight categories. There was also no clear linear relationship between the weight limit in a category and the percentage of defended brings (rS(4942) = −0.04, *p* = 0.003).

## 4. Discussion

The length of fight time to submission finish remains consistent across all weight divisions. Fighters in the heaviest categories experienced significantly shorter fights compared to those in lighter weight classes. A weak but statistically significant negative correlation was observed between the weight limit of a category and fight duration in men, indicating that the heavier the weight category, the shorter the fight time. However, while this pattern did not reach significance in women, a gender-based comparison revealed that female bouts were approximately one and a half minutes longer on average than male bouts, and women’s fights had significantly fewer submission attempts compared to men’s fights. Specifically, women recorded an average of 0.32 submission attempts, compared to men’s average of 0.45 submission attempts. Fewer submission attempts might indicate a different approach or fighting style, possibly reducing cumulative exposure to the dangerous grip or chokehold techniques. On the other hand, even infrequent but high-risk moves can result in serious injuries, highlighting the importance of training fighters in safe practice techniques and effective defense against submissions. However, when examining submission attempts within the same weight category, these gender differences were not observed, indicating that factors related to weight class play a role in the frequency of submission techniques used. Both the fighter’s characteristics and the assigned weight class influence the way fights progress and the outcomes regarding submissions and overall duration. Most submissions occurred in the first round of the fight, which accounted for 52.20% of the cases.

The most common submission technique in evaluated fights was a rear-naked choke. 68.6% of all submissions were caused by chokes, more frequently in male than female fights. Similar results were presented by Stellpflug et al. [15], who analyzed all fight-ending chokes in the UFC. During the evaluated period, there were 904 chokes, which ended 15.5% of fights, making it the most common submission technique—76.2%. Most chokes were ended by voluntary stoppage by a fighter, but 11% of them resulted in loss of consciousness. Neck compression used to end a fight involves squeezing the carotid arteries and the jugular veins to lower cerebral perfusion pressure and reduce blood flow to the brain [16,17,18]. On average, after ten seconds of such constrictions, the fighter loses consciousness. Sportive chokes are considered safe, especially when surveying the fighters themselves [19]. However, safety precautions are necessary as there were dangers connected to chokes in combat sports reported [20]. Hubbard et al. [20] stated that near chokes, chokes, and submission holds in MMA may result in temporary anoxic brain injury leading to similar changes that occur during a concussive injury. High incidence of chokes and submission by this type of technique should be considered in the care plan of MMA athletes.

The types of injuries sustained in mixed martial arts depend on the techniques allowed during a competition. A prospective study on various combat sports participants showed that those training in wrestling had high rates of joint injuries [21]. Many studies show that while striking techniques lead to head and facial trauma, submission techniques are detrimental to joints [22,23,24,25]. A study evaluating the incidence of injury in BJJ competitions shows that there are 9.2 injuries per 1000 exposures, and the most common are orthopedic ones—78% of all [25]. The most frequently injured joint during submission techniques is the elbow, with the hyperextension injury due to armbar being the most common mechanism. In our study, arm- and hand-concentrated submission techniques, including armbar, accounted for 27.1% of submissions. The armbar caused the end of 63 out of all evaluated fights. The injury rates in MMA are estimated to be 236–286 per 1000 athlete-exposure hours—higher than in BJJ [4,26]. The observational cohort study of MMA competitions showed that the most common injuries were abrasions, lacerations, and concussions [26]. There were, however, 11 orthopedic injuries noted among 232 exposures. Most MMA competition ends with a decision or submission [4]. The athletes’ and referees’ cautiousness and knowledge about the risk of sustaining an injury during the submission attempts should be considered as crucial preventive measures. Additionally, flexibility, balance, and stability training are crucial for preventing grappling injury [27].

Although extensive research has been conducted on the injury type in MMA and BJJ, there is still not enough knowledge about the mechanism connected with specific submission techniques [28,29]. While studies report general injury rates and common anatomical sites, only a few of them provide detailed information. The injury preventive methods include education aiming at increasing awareness and medical supervision [30,31,32]. However, there is still a lack of sufficient knowledge among the athletes, and some techniques perceived as low risk, such as chokeholds, have been reported in case reports of severe vascular injuries, including cervical artery dissections and ischemic strokes [30,31,33]. The armlock, a technique where forced hyperextension of the elbow is applied using the hip as a pivot and body weight a leverage, has been reported to cause flexor pronator mass injury, medial collateral ligament tears, mild edema, and capsule irregularities [34]. There has also been cerebral infarct with complete occlusion of the vertebral artery secondary to dissection reported as grappling injury in MMA [35]. Recurrent takedowns and submission techniques may also lead to cumulative joint stress and thus it is important to discuss standardized injury prevention protocols.

The strong point of the presented research is that it included a high number of fights, and it is the first study to include both professional male and female fighters. Comparing different fight characteristics between weight categories was also a strong feature of the conducted study. However, in some aspects of the research, female fights and certain weight classes could not be considered in the evaluation due to the lower number of participants, which would have led to mistakes in statistical interpretation. Further evaluation of these groups in a higher number of fights would be beneficial in the future. Among the limitations, it also worth noting that the retrospective nature of this evaluation may not provide sufficient information. Although the submission technique that led to the end of the fight was evaluated and verified, it is worth noting that the final technique may not be the most crucial factor in determining the fight result. Through retrospective analysis, we may only suspect that it had the greatest influence on the outcome. The methodology applied in this study assumes an association between fight termination by submission and the risk of injury, without a subsequent assessment of the percentage of fights that resulted in an injury. Due to this aspect, conducting further prospective clinical research to evaluate the incidence of injuries among fighters, including direct clinical injury data, diagnosis, and outcomes, should be considered.

## 5. Conclusions

MMA fights frequently end by submission, and many submissions involve techniques that put considerable stress on the joints and cervical region. Fighters and referees need clear protocols to swiftly recognize when a submission poses excessive risk, such as when a choke leads to loss of consciousness, which is a critical indicator of potential harm. Training programs focusing on flexibility, balance, and stability are also important in preventing such injuries during a fight. Additionally, educating fighters on the frequency, mechanics, and risks of each submission technique can help them better understand and possibly avoid or quickly tap out from dangerous holds, reducing the likelihood of severe injuries.

## Figures and Tables

**Table 1:** Comparison of fight results depending on the sex of the fighter.

Result	Male	Female	Total
Unanimous decision	35.6% (820)	41.4% (70)	36% (890)
Split decision	9.1% (209)	14.8% (25)	9.5% (234)
KO/TKO *	36.5% (841)	26% (44)	35.8% (885)
Submission	18.8% (432)	17.8% (30)	18.7% (462)
Total	2302	169	2471

* KO/TKO—knockout/technical knockout.

**Table 2:** Comparison of fight results depending on the sex of the fighter among title and non-title fights.

Result	Title Fights	Non-Title Fights	Total
Unanimous decision	31.3% (81)	36.6% (809)	36% (890)
Split decision	5.4% (14)	9.9% (220)	9.5% (234)
KO/TKO *	45.9% (119)	34.6% (766)	35.8% (885)
Submission	17.4% (45)	18.9% (417)	18.7% (462)
Total	259	2212	2471

* KO/TKO—knockout/technical knockout.

**Table 3:** Comparison of fight results depending on the weight category.

	*n*	Unanimous Decision	Split Decision	KO/TKO *	Submission
FLYWEIGHT	72	53% (38)	11% (8)	18% (13)	18% (13)
BANTAMWEIGHT	170	41% (69)	11% (18)	30% (51)	19% (32)
FEATHERWEIGHT	182	49% (89)	13% (23)	29% (53)	9% (17)
LIGHTWEIGHT	439	37% (164)	12% (51)	29% (125)	23% (99)
WELTERWEIGHT	481	38% (183)	10% (46)	34% (163)	19% (89)
MIDDLEWEIGHT	354	31% (111)	8% (28)	36% (129)	24% (86)
LIGHT HEAVYWEIGHT	302	30% (91)	8% (25)	45% (135)	17% (51)
HEAVYWEIGHT	285	23% (66)	3% (9)	59% (167)	15% (43)
CATCH WEIGHT BOUT	17 *	53% (9)	6% (1)	29% (5)	12% (2)
WOMEN STRAWWEIGHT	65	48% (31)	15% (10)	19% (12)	19% (12)
WOMEN FLYWEIGHT	36	50% (18)	14% (5)	25% (9)	11% (4)
WOMEN BANTAMWEIGHT	57	28% (16)	18% 10	33% (19)	21% (12)
WOMEN FEATHERWEIGHT	11 *	46% (5)	0%	36% (4)	18% (2)
Total	2471	36% (890)	10% (234)	36% (885)	19% (462)

* KO/TKO—knockout/technical knockout.

**Table 4:** Type of fight depending on the sex of the athlete.

Type of Fight	Man	Woman	Total
Title fights	9.8% (227)	20.1% (34)	10.5% (261)
Non-title fights	90.2% (2092)	79.9% (135)	89.5% (2227)
Total	2319	169	2488

**Table 5:** Duration of fight depending on the weight category.

	Weight Category	*n*	Duration of Fight	
			M	SD
MALE FIGHTS	FLYWEIGHT	72	12 min 14 s	6 min 4 s
	BANTAMWEIGHT	171	11 min 36 s	5 min 56 s
	FEATHERWEIGHT	182	12 min 50 s	5 min 38 s
	LIGHTWEIGHT	441	10 min 50 s	6 min
	WELTERWEIGHT	484	10 min 44 s	6 min 7 s
	MIDDLEWEIGHT	358	9 min 55 s	6 min
	LIGHT HEAVYWEIGHT	305	9 min 29 s	6 min 22 s
	HEAVYWEIGHT	289	8 min 27 s	6 min 14 s
	CATCH WEIGHT BOUT	17	11 min 7 s	6 min 45 s
FEMALE FIGHTS	STRAWWEIGHT	65	12 min 27 s	6 min 10 s
	FLYWEIGHT	36	13 min 7 s	4 min 35 s
	BANTAMWEIGHT	57	11 min 5 s	6 min 27 s
	FEATHERWEIGHT	11	11 min 39 s	10 min 2 s
TOTAL		2488	10 min 34 s	6 min 12 s

**Table 6:** Percentage of fights of a particular length (in rounds) ended by submission according to the gender of the fighters.

	Male Fights		Female Fights		Total	
Rounds	*n*	%	*n*	%	*n*	%
1	223	51.60%	18	60.00%	241	52.20%
2	135	31.30%	5	16.70%	140	30.30%
3	66	15.30%	6	20.00%	72	15.60%
4	4	0.90%	0	0.00%	4	0.90%
5	4	0.90%	1	3.30%	5	1.10%
Total	432	100.00%	30	100.00%	462	100.00%

**Table 7:** Percentage of fights of each length (in rounds) completed by submission according to whether the fights were title fights or not.

	Non-Title Fights		Title Fights		Total	
Rounds	*n*	%	*n*	%	*n*	%
1	227	54.40%	14	31.10%	241	52.20%
2	128	30.70%	12	26.70%	140	30.30%
3	62	14.90%	10	22.20%	72	15.60%
4	0	0.90%	4	8.90%	4	0.90%
5	0	0.90%	5	11.10%	5	1.10%
Total	417	100.00%	45	100.00%	462	100.00%

**Table 8:** Percentage of fights completed by submission in a given number of rounds according to the weight category of the fight.

	Rounds					Total
	1	2	3	4	5	
BANTAMWEIGHT	43.8% (14)	37.5%(12)	18.8% (6)	0	0	32
CATCH WEIGHT BOUT	50% (1)	0	50% (1)	0	0	2
FEATHERWEIGHT	58.8% (10)	5.9% (1)	35.3% (6)	0	0	17
FLYWEIGHT	46.2% (6)	15.4% (2)	23.1% (3)	0	15.4% (2)	13
HEAVYWEIGHT	69.8% (30)	20.9% (9)	9.3% (4)	0	0	43
LIGHT HEAVYWEIGHT	51% (26)	37.3% (19)	7.8% (4)	3.9% (2)	0	51
LIGHTWEIGHT	47.5% (47)	33.3% (33)	16.2% (16)	2% (2)	1% (1)	99
MIDDLEWEIGHT	52.3% (45)	31.4% (27)	15.1% (13)	0	1.2% (1)	86
WELTERWEIGHT	49.4% (44)	36% (32)	14.6% (13)	0	0	89
WOMEN BANTAMWEIGHT	41.7% (5)	25% (3)	25% (3)	0	8.3% (1)	12
WOMEN FLYWEIGHT	25% (1)	50% (2)	25% (1)	0	0	4
WOMEN’S FEATHERWEIGHT	100% (2)	0	0	0	0	2
WOMEN’S STRAWWEIGHT	83.3% (10)	0	16.7% (2)	0	0	12
Total	52.2% (241)	30.3% (140)	15.6% (72)	0.9% (4)	1.1% (5)	462

**Table 9:** Duration of fights ended by submission according to weight category.

	M	SD	*n*
BANTAMWEIGHT	6 min 26 s	4 min 3 s	32
CATCH WEIGHT BOUT	6 min 4 s	6 min 41 s	2
FEATHERWEIGHT	6 min 52 s	4 min 38 s	17
FLYWEIGHT	6 min 50 s	6 min 23 s	13
HEAVYWEIGHT	4 min 23 s	3 min 22 s	43
LIGHT HEAVYWEIGHT	5 min 57 s	4 min 4 s	51
LIGHTWEIGHT	6 min 41 s	4 min 34 s	99
MIDDLEWEIGHT	6 min	4 min 26 s	86
WELTERWEIGHT	6 min 17 s	3 min 43 s	89
WOMEN BANTAMWEIGHT	8 min 45 s	6 min 18 s	12
WOMEN FLYWEIGHT	7 min 14 s	3 min 2 s	4
WOMEN’S FEATHERWEIGHT	3 min	1 min 21 s	2
WOMEN’S STRAWWEIGHT	4 min 47 s	3 min 43 s	12
Total	6 min 10 s	4 min 20 s	462

**Table 10:** Fight control time (in seconds) depending on weight categories, sex, and fight result (F1–fighter who won; F2—fighter who lost).

		F1			F2			TOTAL		
		*n*	M	SD	*n*	M	SD	*n*	M	SD
MALE FIGHTS	FLYWEIGHT	72	228	246	72	80	108	144	154	203
	BANTAMWEIGHT	171	150	163	171	72	110	342	111	144
	FEATHERWEIGHT	182	195	198	182	89	115	364	142	170
	LIGHTWEIGHT	441	202	223	441	85	115	882	143	186
	MIDDLEWEIGHT	358	178	191	358	91	128	716	134	168
	WELTERWEIGHT	484	236	240	484	83	111	968	159	202
	LIGHT HEAVYWEIGHT	305	186	225	305	62	100	610	124	185
	HEAVYWEIGHT	289	168	201	289	76	114	578	122	170
	CATCH WEIGHT BOUT *	17	153	156	17	69	107	34	111	139
FEMALE FIGHTS	STRAWWEIGHT	65	158	166	65	105	129	130	132	150
	FLYWEIGHT *	36	202	201	36	108	121	72	155	171
	BANTAMWEIGHT	57	197	213	57	94	131	114	146	183
	FEATHERWEIGHT *	11	148	216	11	103	155	22	126	185

Caution: * indicates categories not included in the analysis of variance.

**Table 11:** Submission techniques resulting in fight ending depending on weight categories.

Submission Technique	Bantamweight	Catch Weight	Featherweight	Flyweight	Heavyweight	Light Heavyweight	Light Weight	Middleweight	Welterweight	Women Bantam Weight	Woman Fly Weight	Women Feather Weight	Women Strawweight	Total
Anaconda Choke	1		1			3		1	3					**9**
Ankle Lock					1		1	1	1					**4**
Arm Triangle				1	5	4	1	7	7	1				**26**
Armbar	3		1	2	9	6	10	5	14	6	1		6	**63**
Bulldog Choke	1						1		3	1				**6**
D’Arce Choke	1				2		4	2	4					**13**
Decision by referee								1						**1**
Ezekiel Choke					1									**1**
Flying Triangle			1											**1**
Guillotine Choke	9		6	5	5	8	19	22	14				1	**89**
Heel Hook			1		2		1	3						**7**
Jaw Injury							1							**1**
Knee Injury									1					**1**
Shoulder Injury									1					**1**
Inverted Triangle							1							**1**
Keylock						1								**1**
Kimura				1	3	4	5	2	6					**21**
Kneebar	1				1		1		2					**5**
Control Body Triangle								1						**1**
Shoulder Lock					1									**1**
Neck Crank					1	1	1	1						**4**
North–South Choke					1				1					**2**
Rear-Naked Choke	14	1	4	4	7	20	42	26	25	4	2		5	**154**
Scarf Hold					1									**1**
Schultz Front Headlock									1					**1**
Straight Armbar					1									**1**
Suloev Stretch	1		1						1					**3**
Toe Hold					1									**1**
Triangle Armbar			1				1	1			1	1		**5**
Triangle Choke	1	1	1		1	2	10	13	5			1		**35**
Von Flue Choke						2								**2**
**Total**	**32**	**2**	**17**	**13**	**43**	**51**	**99**	**86**	**89**	**12**	**4**	**2**	**12**	**462**

**Table 12:** Frequency of chokes or arm/hand techniques submission by weight category.

	Chokes	Arm/Hand Techniques	Total
BANTAMWEIGHT	81.3% (26)	12.5% (4)	32
CATCH WEIGHT BOUT	100% (2)	0	2
FEATHERWEIGHT	76.5% (13)	11.8% (2)	17
FLYWEIGHT	69.2% (9)	30.8% (4)	13
HEAVYWEIGHT	44.2% (19)	48.8% (21)	43
LIGHT HEAVYWEIGHT	70.6% (36)	29.4% (15)	51
LIGHTWEIGHT	75.8% (75)	20.2% (20)	99
MIDDLEWEIGHT	76.7% (66)	17.4% (15)	86
WELTERWEIGHT	62.9% (56)	32.6% (29)	89
WOMEN BANTAMWEIGHT	41.7% (5)	58.3% (7)	12
WOMEN FLYWEIGHT	50% (2)	50% (2)	4
WOMEN’S FEATHERWEIGHT	50% (1)	50% (1)	2
WOMEN’S STRAWWEIGHT	58.3% (7)	41.7% (5)	12
**TOTAL**	68.6% (317)	27.1% (125)	462

**Table 13:** Percentage of defended takedowns in men’s and women’s fights by weight category.

		M	SD	*n*
**MALE FIGHTS**	FLYWEIGHT	31%	35%	144
	BANTAMWEIGHT	27%	36%	342
	FEATHERWEIGHT	27%	35%	364
	LIGHTWEIGHT	28%	36%	882
	MIDDLEWEIGHT	28%	36%	716
	WELTERWEIGHT	29%	37%	968
	LIGHT HEAVYWEIGHT	25%	36%	610
	HEAVYWEIGHT	26%	38%	578
	CATCH WEIGHT BOUT	40%	43%	34
**FEMALE FIGHTS**	STRAWWEIGHT	29%	37%	130
	FLYWEIGHT	32%	37%	72
	BANTAMWEIGHT	28%	37%	114
	FEATHERWEIGHT	15%	30%	22
	Total	28%	36%	4976

## Data Availability

Data supporting this study are available online on http://ufcstats.com/statistics/events/completed (accessed on 27 July 2025). Evaluated data are available on reasonable request from corresponding author.
